# A Novel Peritoneal Dialysis Fluid Based on Succinylated Gelatin and Citrate: A Preliminary Investigation of Efficacy, Safety, and Biocompatibility

**DOI:** 10.3390/ph19020222

**Published:** 2026-01-27

**Authors:** Qing Xu, Zhifeng Zhou, Yi Zheng, Lu Jin, Chen Liu, Peiyun Li, Fang Wang, Ping Fu, Ling Zhang

**Affiliations:** Department of Nephrology, Kidney Research Institute, West China Hospital, Sichuan University, Chengdu 610041, China; xvqingscu@163.com (Q.X.); zhouzf97@163.com (Z.Z.); zhengyi@wchscu.edu.cn (Y.Z.); yiqinggou@163.com (L.J.); chenliu@scu.edu.cn (C.L.); peiyunli123@163.com (P.L.); hxwf11@163.com (F.W.); fupinghx@scu.edu.cn (P.F.)

**Keywords:** peritoneal dialysis, PD fluid, biocompatibility, succinylated gelatin, peritoneal membrane

## Abstract

**Introduction:** The metabolic complications and poor biocompatibility of conventional glucose-based (GLU) peritoneal dialysis fluid (PDF) have driven the need for improved alternatives. To address this, we developed and evaluated a novel PDF utilizing succinylated gelatin (GEL) as osmotic agent and citrate as buffer, designed to provide effective solute clearance while offering enhanced biocompatibility. **Methods:** Physicochemical parameters (pH and osmolality) of the novel GEL-PDF were measured. Its performance was assessed in rats with chronic kidney disease. A total of 20 rats were randomized into short-term experiments to evaluate 4 h creatinine clearance and ultrafiltration (UF). A 12-week long-term experiment (*n* = 35) compared the GEL-PDF against normal saline (NS), GLU, and icodextrin-based (ICO) PDFs, monitoring survival, biochemical parameters, peritoneal membrane histology, and kidney histology. **Results:** The GEL-PDF demonstrated a neutral pH (7.30) and lower osmolality (317 mOsm/L) compared to GLU-PDF. In the short-term experiment, GEL-PDF achieved effective creatinine clearance by 4 h and provided higher 4 h UF than NS and GLU, comparable to ICO. However, during prolonged dwells (6–16 h), its UF was inferior to ICO. In the long-term experiment, GEL-PDF preserved peritoneal membrane structure, showing the least thickness and collagen deposition. Furthermore, the GEL-PDF demonstrated superior preservation of serum albumin compared to the GLU-PDF. It also exhibited a more favorable lipid profile, as evidenced by significantly lower total cholesterol levels than the ICO group at 12 weeks (*p* = 0.035), with no adverse effects on electrolytes, liver function, or glucose metabolism. **Conclusions:** The novel GEL and citrate-based PDF provide effective short-dwell UF and solute removal while exhibiting superior biocompatibility, as evidenced by significant protection against peritoneal membrane injury and favorable metabolic profiles. Although its long-duration UF was lower than that of ICO, it substantially outperformed GLU-PDF. These properties position the GEL-PDF as a promising candidate for short- to medium-dwell exchanges, particularly for daytime use, where it could fill an important clinical gap by providing enhanced UF without the high GLU exposure associated with conventional PDF.

## 1. Introduction

Peritoneal dialysis (PD) is a critical home-based renal replacement therapy (RRT) for patients with end-stage kidney disease (ESKD). In 2018, approximately 11% of maintenance dialysis patients received PD. More than half of the PD population resided in China, Mexico, United States, and Thailand [1]. Additionally, the use of PD has grown rapidly in these countries. The increasing prevalence of ESKD against a backdrop of constrained healthcare resources underscores the value of PD as a resource-efficient modality [2].

Currently, commercially available peritoneal dialysis fluids (PDFs) include glucose-based (GLU), amino acid-based, and icodextrin-based (ICO) PDFs. Currently, GLU-PDFs remain the clinical mainstay due to their cost-effectiveness and accessibility. However, their poor biocompatibility—driven by low pH, high glucose exposure, and glucose degradation products (GDPs)—induces peritoneal oxidative stress, inflammation, and fibrosis [3,4,5]. Additionally, systemic glucose absorption contributes to ultrafiltration (UF) failure and metabolic complications such as dyslipidemia and insulin resistance [6,7,8].

Although neutral-pH, low-GDP GLU solutions represent an advancement, they still rely on high glucose concentrations for osmosis and thus fail to address the fundamental issue of glucose-mediated toxicity [9,10,11,12]. While alternative PDFs exist, including amino acid-based and icodextrin-based (ICO) solutions, they can only be consumed in restricted doses. Amino acid-based PDFs may exacerbate metabolic acidosis and increase nitrogen load, potentially causing gastrointestinal side effects. Consequently, they are recommended only once daily for malnourished patients. ICO, valued for its superior long-dwell UF performance, is advised only for a single nocturnal long dwell. Furthermore, its metabolite maltose can accumulate systemically and interfere with certain blood glucose monitoring systems, leading to spuriously high glucose readings and the risk of inappropriate insulin administration. Furthermore, these glucose-sparing PDFs retain the conventional lactate buffer—which itself may promote peritoneal fibrosis [13,14,15,16,17]. Above all, these glucose-free alternatives are each recommended only once daily. In current practice, patients therefore still require concomitant use of GLU-PDF for other exchanges, underscoring an unmet need for a formulation that could broadly replace GLU-PDF while offering a more biocompatible buffer system.

To address this specific gap, we developed a novel PDF that innovates on both the osmotic agent and the buffer system [18]. Succinylated gelatin (GEL) is a colloidal agent with high molecular weight (20–37 kDa), acting as an osmotic driver while restricting peritoneal absorption. Previous studies have demonstrated that citrate can improve UF and creatinine (Cre) clearance during PD, while also extending the lifespan of PD catheters [19,20]. In terms of safety, citrate excretion is not dependent on the kidney. Crucially, citrate metabolism (primarily hepatic conversion to bicarbonate with calcium release) is renal-independent, making it suitable for end-stage renal disease (ESRD) patients. The distinct advantages of the GEL–citrate PDF are as follows: (1) its synergistic innovation targets both the osmotic agent and buffer to enhance biocompatibility; (2) GEL is used as a colloidal osmotic agent, which provides more sustained and stable UF than crystalloid agents, avoiding the metabolic complications associated with peritoneal absorption of glucose or amino acids. (3) In contrast to ICO, which is tailored for prolonged nocturnal dwells, GEL is expected to provide sustained mild UF suitable for daytime exchanges. This positions it to address the critical clinical gap for a glucose-free option during frequent daytime dwells, where ICO is not indicated and GLU-PDF remains the default.

In summary, we formulated a novel PDF by combining GEL with citrate and conducted a preliminary investigation into its efficacy, safety, and biocompatibility, with the aim of expanding future clinical options.

## 2. Results

### 2.1. Physicochemical Properties of the Included Peritoneal Dialysis Fluids

The physicochemical properties of the tested PDFs are summarized below. The pH values were 6.85, 5.37, 7.05, and 7.30 for the NS, GLU, ICO, and GEL-PDFs, respectively. Corresponding osmolality values were 263, 340, 287, and 317 mOsm/L. Furthermore, the GLU and ICO-PDFs were buffered with 40 mmol/L lactate, whereas the GEL-PDF utilized 14 mmol/L citrate as its buffer. According to manufacturers, the molecule weight of GEL ranges from 20 to 37 k Dalton. A detailed comparison of these properties is provided in Table 1.

### 2.2. Short-Term Animal Experiments

#### 2.2.1. Temporal Changes in Creatinine D/P (Dialysate-to-Plasma) Ratio in the GEL Group

**Figure 1:**
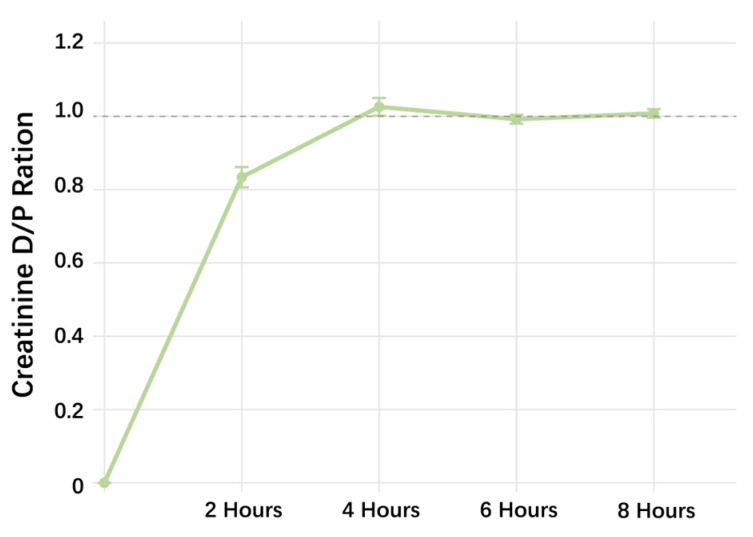
Time-dependent changes in creatinine D/P ratio of GEL-PDF (*n* = 5).

To evaluate the dialysis efficacy of the novel GEL-PDF, 30 mL dialysate was administered intraperitoneally to each rat. Effluent samples (0.5–1 mL) were collected at 2, 4, 6, and 8 h, and Cre levels were measured. The results showed that the Cre concentration in the effluent peaked at 4 h and remained stable throughout the remaining dwell time (Figure 1).

#### 2.2.2. Ultrafiltration Capacity of Different Peritoneal Dialysis Fluids

**Figure 2:**
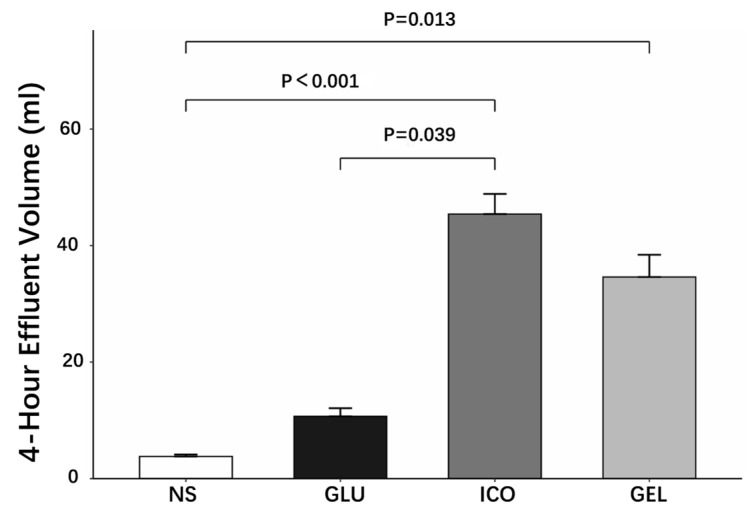
Drainage volumes after a 4 h dwell (*n* = 5 per group).

The drainage volumes after 4 h dwell varied significantly among the four PDFs. The ICO demonstrated the highest drainage volume (45.4 ± 7.8 mL), followed by the GEL (34.6 ± 8.6 mL), while the GLU and NS showed lower volumes (10.7 ± 3.1 mL and 3.8 ± 0.8 mL, respectively). Specifically, both colloidal PDFs (ICO and GEL) showed higher drainage volumes compared to NS (ICO vs. NS: *p* < 0.001; GEL vs. NS: *p* = 0.013). No significant difference was observed between the two colloidal PDFs (ICO vs. GEL: *p* = 1.000) (Figure 2). These findings indicate that the colloidal osmotic agents provide significantly enhanced UF capability compared to crystalloid PDFs during 4 h dwell.

Further investigations compared the long-term UF capacity (6, 8, 16 h) between ICO and GEL. Across all evaluated time points, ICO consistently exhibited greater UF capacity than GEL, with statistically significant differences at 6 (*p* < 0.001) and 16 h (*p* = 0.006), and a marginal difference at 8 h (*p* = 0.071).

### 2.3. Long-Term Animal Experiments

The long-term experiment consisted of five rats in the NS group and ten rats in each of the GLU, ICO, and GEL groups, in which all animals received 30 mL intraperitoneal infusion of respective PDFs per day over 12 weeks. The survival outcomes were monitored throughout the study. The NS group maintained full survival (5/5) throughout the 12-week study. In the GLU group, six out of ten rats survived, with mortalities occurring primarily during the first 4 weeks. The ICO group exhibited no mortality in the first 8 weeks, but three deaths were observed in the final phase, resulting in final survival of 7/10. In the GEL group, a steady attrition was observed, with one death in each phase and a final survival of 7/10. Formal statistical comparisons of survival were not performed. These descriptive findings should be interpreted with caution.

#### 2.3.1. Changes in Rat Blood Parameters over the Dialysis Period

During the 12-week PD period, blood was drawn every 4 weeks for laboratory testing. The assessed parameters included the following: renal function (Scr and BUN), liver function (ALT, AST, ALP), electrolytes (K^+^, Na^+^, Ca^2+^, iCa, Cl^−^), lipid profile (TC, LDL), ALB, TP, and phosphorus (Supplementary Materials, Table S1). Additionally, GSP, random blood glucose, and Lac were assessed at the terminal point (Table 2).

##### Renal Function: Serum Creatinine and Blood Urea Nitrogen

**Figure 3:**
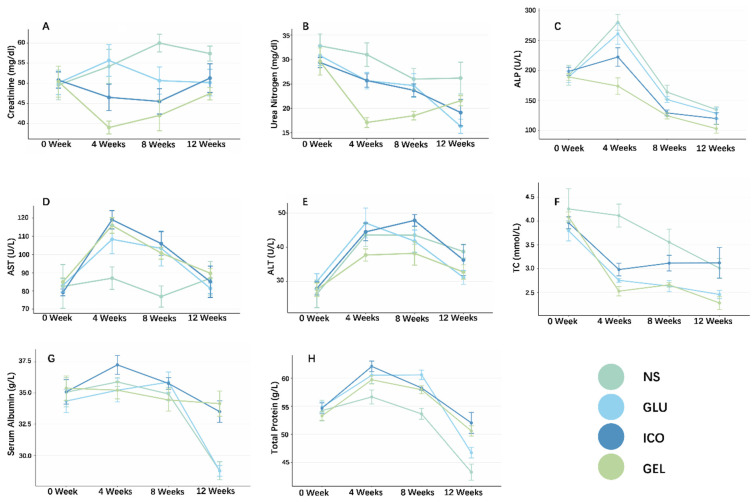
(**A**–**H**) Blood biochemical parameter change during long-term experiment (sample sizes at each time point were as follows: baseline—NS:5, GLU:10, ICO:10, GEL:10; 4 weeks—NS:5, GLU:7, ICO:10, GEL:9; 8 weeks—NS:5, GLU:7, ICO:10, GEL:8; 12 weeks—NS:5, GLU:6, ICO:7, GEL:7. The detailed information, including *p*-values, are provided in the Supplementary Materials, Table S1).

Initial Scr levels were comparable across all four groups (*p* = 0.996). During the first 4 weeks, the GEL group exhibited a reduction in Scr, with levels significantly lower than those in the Glu (*p* = 0.004) and NS (*p* = 0.023) groups. Following 8 weeks of PD, Scr levels in both the GEL and ICO groups were lower than that in the NS group (*p* = 0.017 and *p* = 0.044, respectively). However, these two groups no longer demonstrated superiority in Scr levels over the Glu group. By the end of the experiment, the previously observed intergroup differences in Scr levels for the Gel and ICO groups over the other two groups were no longer statistically significant (*p* = 0.126). Throughout the experiment, Scr levels did not differ significantly between the GEL and ICO groups (Figure 3A).

At baseline, BUN did not differ among the four groups (*p* = 0.769). Results revealed that the BUN level in the GEL group decreased rapidly during the first 4 weeks (NS vs. GEL: *p* < 0.001, GLU vs. GEL: *p* = 0.021, ICO vs. GEL: *p* = 0.006), a trend similar to that of Scr. However, this advantage of GEL PDF gradually diminished over the course of the experiment. By week 8, the BUN levels in the GEL group remained lower than that in the NS group (*p* = 0.026), but the differences compared to the GLU and ICO groups narrowed (GLU vs. GEL: *p* = 0.062, ICO vs. GEL: *p* = 0.078). By week 12, no significant differences in BUN levels were detected between the GEL group and the other three groups (Figure 3B).

##### Liver Function

Liver function during experiment was assessed within three parameters: ALP, AST, and ALT. After the establishment of the rat CKD model, baseline biochemical parameters were measured, revealing no statistically significant differences in the three parameters among the groups. Liver function was subsequently evaluated every four weeks. The results showed that after 4 weeks of PD, the ALP levels in the GEL group were significantly lower than those in the NS and GLU groups (NS vs. GEL: *p* < 0.001; GLU vs. GEL: *p* = 0.004). At the 8-week mark, the differences, though slightly attenuated, remained statistically significant (NS vs. GEL: *p* = 0.002; GLU vs. GEL: *p* = 0.030). Subsequently, the intergroup disparities continued to lessen. By the end of the study, only a marginal difference in ALP levels was observed between the GEL and NS groups (*p* = 0.078). Throughout the entire experimental period, no significant difference in ALP levels was detected between the ICO and GEL groups (Figure 3C).

Throughout the study, AST levels in the GLU, ICO, and GEL groups exhibited an initial increase within the first 4 weeks, followed by a subsequent decline until the endpoint. No statistically significant differences in AST levels were observed among these three groups at any time point. Similarly to the AST profile, ALT levels exhibited a transient rise in the first 4 weeks before gradually decreasing, albeit with a less pronounced amplitude. No statistically significant differences in ALT were observed among any of the groups over the experimental period (Figure 3D,E).

##### Blood Lipids, Serum Albumin, Total Protein and Phosphorus

Throughout the study, the GEL group demonstrated comparable efficacy to the GLU group in TC levels. Notably, the GEL group showed lower TC levels than the ICO group at both week 4 (*p* = 0.049) and week 12 (*p* = 0.035) (Figure 3F). The NS group consistently exhibited the highest TC levels among all groups. No significant intergroup differences were observed in LDL levels at any time point.

After 12 weeks, serum ALB levels in the ICO and GEL groups were significantly higher than those in the other two groups (NS vs. ICO: *p* = 0.011, NS vs. GEL: *p* = 0.002; Glu vs. ICO: *p* = 0.007, Glu vs. GEL: *p* = 0.001) (Figure 3G), indicating that the colloidal osmotic agents ICO and GEL could effectively reduce transperitoneal ALB loss.

Additionally, the type of PDF also influenced TP levels in rats. At the end of the experiment, TP in both colloidal PDF groups were higher than those in the NS group (NS vs. ICO: *p* = 0.001, NS vs. GEL: *p* = 0.008) (Figure 3H). However, this difference did not achieve statistical significance when compared to the GLU group.

Throughout the experiment, serum phosphorus levels demonstrated a gradual declining trend across all groups during the first 8 weeks, with no significant intergroup differences. However, in the 12 weeks, the NS group showed a rebound in phosphorus levels, resulting in statistically significant differences when compared to both the ICO and GEL groups (ICO vs. NS *p* = 0.034; NS vs. GEL *p* = 0.047).

##### Electrolyte

Serum electrolytes were regularly monitored every 4 weeks throughout the experiment. Although statistically significant differences in Na^+^ and Cl^−^ levels were observed, the actual variations represented normal physiological fluctuations and were deemed clinically insignificant. No significant intergroup differences were detected in iCa^2+^ or total Ca^2+^ levels. At baseline, K^+^ in the GEL group was higher compared to the other three groups (*p* < 0.001). However, as the experiment progressed, K^+^ levels became comparable across all groups, with no sustained significant differences observed thereafter.

##### Other Blood Parameters at the End of the Experiment

To assess metabolic outcomes, GSP, Lac, and random blood glucose levels were measured after 12 weeks of dialysis. Statistical analysis indicated no significant differences among the groups in GSP (*p* = 0.173), lactate (*p* = 0.150), or random blood glucose (*p* = 0.866).

#### 2.3.2. Evaluation of Peritoneal Effluent After 12 Weeks of Dialysis

On the final day of the 12-week experiment, rats were infused with 30 mL of warmed PDF and euthanized after a 4 h dwell for effluent collection. Analysis of the drainage volumes revealed significant differences among the groups. The ICO-PDF demonstrated the highest drainage volume (48.0 ± 4.9 mL), significantly higher than both the GLU (1.7 ± 1.9 mL, *p* < 0.001) and NS (10.0 ± 4.6 mL, *p* = 0.007) groups. The GEL-PDF showed intermediate UF (28.6 ± 8.1 mL), which was significantly superior to the GLU-PDF (*p* = 0.017) but not statistically different from ICO (*p* = 0.225).

Biochemical testing of the 4 h peritoneal effluent composition revealed comparable transport of small solutes across all groups, as indicated by the D/P ratios for Cre and BUN (Table 3). Among the active PDFs (GLU, ICO, GEL), BUN D/P ratios were similar, and the GEL group showed a higher ratio compared to the NS group (*p* = 0.062), suggesting its potential for superior urea nitrogen removal. Similarly, effluent electrolyte levels—including iCa, Na^+^, Cl^−^, and K^+^—remained stable and comparable among the groups, with the exception of a lower K^+^ level in the ICO group compared to NS (*p* = 0.030). Furthermore, the effluent in the GEL group exhibited the lowest ALB concentration (4.5 ± 2.4 g/L), positioning it as a promising fluid for reducing transperitoneal ALB loss, although this difference did not reach statistical significance (*p* = 0.507).

#### 2.3.3. Long-Term Impact on Peritoneal Membrane Pathology

**Figure 4:**
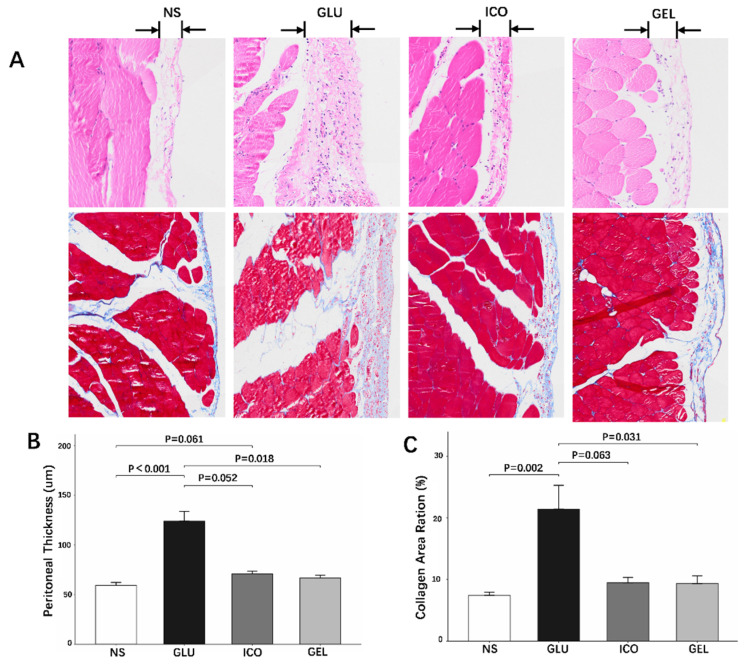
(**A**–**C**) Peritoneal membrane structure after long-term experiment: HE and Masson staining. 20× magnification (Masson staining: collagen fibers are stained blue, *n* = five per group).

Histological assessment of peritoneal thickness at 12 weeks revealed distinct differences among the groups. The GLU group demonstrated significantly greater peritoneal thickness compared to the NS (*p* < 0.001), ICO (*p* = 0.052), and GEL (*p* = 0.018) groups. In contrast, the GEL group showed no significant difference in thickness compared to the NS group (*p* = 0.092). Notably, the ICO group exhibited a trend toward greater thickness than the NS group, which approached but did not reach statistical significance (*p* = 0.061) (Figure 4A,B).

Quantitative analysis of Masson trichrome-stained sections revealed that both the NS and GEL groups exhibited lower collagen volume fractions compared to the GLU-based dialysate group (NS vs. GLU *p* = 0.002; GEL vs. GLU *p* = 0.031). Additionally, a marginal difference in collagen content was observed between the ICO and GLU groups (*p* = 0.063). These findings indicate that chronic exposure to GLU PDF promotes substantial extracellular matrix accumulation and peritoneal fibrosis (Figure 4A,C).

**Figure 5:**
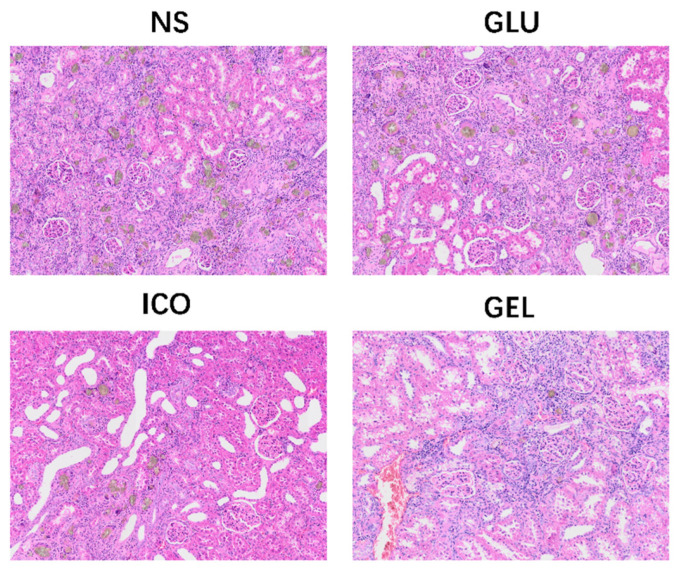
Kidney histology at the end of long-term experiment. 20× magnification (*n* = 3 per group).

Additionally, histological examination of kidney sections at the endpoint revealed adenine crystalline deposition across all groups, confirming the induction of the CKD model. The extent of deposition, however, varied substantially among the groups. Notably, the NS and GLU groups exhibited the most severe adenine accumulation. In contrast, the deposition was markedly attenuated in the ICO and GEL groups, with the GEL group demonstrating the least number of crystalline deposits among all cohorts (Figure 5).

## 3. Discussion

The long-term use of conventional GLU-PDF is complicated by two challenges: peritoneal injury due to poor biocompatibility and metabolic complications. While both amino acid-based and ICO-PDFs offer improved biocompatibility over conventional GLU-PDF, each carries specific limitations. Amino acid-based PDF relies on low molecular weight osmotic agents that undergo rapid transperitoneal absorption, resulting in transient UF. Additionally, they may also induce metabolic acidosis, increase nitrogen load, and cause gastrointestinal discomfort [13]. ICO-PDF provides sustained UF; however, according to manufacturer and health authority recommendations, they are typically prescribed only once daily, necessitating the use of GLU-PDFs for other exchanges. Furthermore, during prolonged dwells, a small amount of ICO-PDF is absorbed systemically and metabolized into maltose, which can interfere with certain glucometers, yielding spuriously high readings and potentially leading to inappropriate insulin administration [14].

With the growing population of PD patients, there are heightened demands for long-term treatment safety and quality of life, making the development and application of novel PDFs that integrate safety, efficacy, and biocompatibility increasingly imperative.

GEL is a colloidal plasma substitute clinically used primarily for treating hypovolemic shock caused by various factors such as hemorrhage, acute trauma, or surgery, burns, and sepsis. It functions by maintaining plasma colloid osmotic pressure, increasing plasma volume, improving venous return and cardiac output, and promoting diuresis. According to pharmacological data from the manufacturers, although GEL is primarily metabolized by the kidneys, its proteolytic degradation demonstrates remarkable adaptability, with no accumulation observed even in patients with renal impairment. Only in hemodialysis patients (Glomeruar filtration rate, GFR < 0.5 mL/min) might the half-life be prolonged. Furthermore, the large molecular weight of GEL (20–37 kDa) limits its peritoneal absorption following intraperitoneal administration, and the minimally absorbed fraction undergoes efficient hepatic metabolism, minimizing the risk of systemic accumulation.

This study systematically developed and evaluated a novel PDF formulated with GEL and citrate. Preliminary evaluation indicates that this GEL-PDF possesses a neutral pH and low osmolarity, demonstrating significant advantages over traditional GLU-PDF. Further short- and long-term animal experiments evaluated the UF, biocompatibility, and safety profile of the novel PDF.

The short-term experiment showed that the Cre level in the effluent peaked after a 4 h dwell. At this time point, GEL also showed good UF, which was significantly higher than that of the NS and GLU, while similar to ICO. A further study compared the long-term UF capacity of the two colloidal PDFs, However, results revealed that the UF of GEL was inferior to ICO during prolonged dwells, which might be related to the partial degradation or absorption of GEL in the peritoneal cavity (Supplementary Materials, Table S2, Figure S1), suggesting that GEL may more suitable for short-to-medium dwell.

The long-term animal experiment further evaluated the safety and biocompatibility of the novel PDF. Results demonstrated that during the first 8 weeks, GEL provided a time-limited enhancement in uremic toxin clearance, as evidenced by reduced Scr and BUN levels in rats, although these differences were no longer statistically significant by week 12.

A key finding of the long-term experiment was the superior capacity of both colloidal PDFs, particularly the novel GEL-PDF, to preserve serum albumin and total protein levels compared to crystalloid PDFs. After 12 weeks PD, rats treated with ICO and GEL exhibited significantly higher serum ALB levels than those in the NS and GLU groups. This was further supported by the analysis of the peritoneal effluent, where the GEL group showed the lowest ALB concentration, indicating a reduction in transperitoneal ALB loss. However, this difference did not reach statistical significance (potentially due to the small sample size). This finding carries significant clinical relevance, as protein–energy wasting and hypoalbuminemia are prevalent and powerful predictors of mortality in ESRD patients [21,22,23,24].

The analysis of lipid metabolism revealed no significant intergroup differences in LDL during the experiment. However, the rats in the GEL group exhibited lower TC levels, with a statistically significant difference compared to the ICO group at 12 weeks. These findings indicate favorable lipid safety for the GEL-PDF and suggest a potential benefit in reducing TC levels. In terms of liver function, the GEL group showed lower ALP levels, while no significant differences were found in other parameters (ALT, AST) among the groups during the entire PD period, indicating that the GEL-PDF does not adversely affect liver function and demonstrates a good safety profile. Throughout the experiment, all three active PDFs (GLU, ICO, and GEL) effectively reduced serum phosphorus levels in rats, and no statistically significant differences were observed among them.

Electrolyte levels remained clinically comparable across all groups. ANOVA identified statistically significant differences in Na^+^ and Cl^−^ levels, and that the absolute differences were minimal (typically 1–3 mmol/L) and remained within the range of normal physiological fluctuation. The statistical significance may reflect low intragroup variability rather than a meaningful difference. Although the baseline serum K^+^ level in the GEL group was higher than other groups. It rapidly normalized and remained within a normal and comparable range throughout the entire 12-week study. In summary, the GEL-PDF demonstrated no adverse effects on electrolyte balance, supporting its favorable safety profile.

To assess the impact of PDFs on glucose metabolism, random blood glucose and GLP levels were measured in all rats at the end of study, with no statistically significant differences observed among the groups. This may be attributed to the fact that only one daily intraperitoneal injection was administered, resulting in minimal glucose exposure even in the GLU group. Moreover, no differences in blood Lac levels were detected among the groups in 12 weeks, further supporting the safety profile of the GEL-PDF.

Evaluation of peritoneal function at the study endpoint revealed that the GEL group exhibited the highest BUN D/P ratio (0.99 ± 0.18), which was marginally higher than that of the NS group (*p* = 0.062) but did not reach statistical significance compared to the other two groups. Conventionally, long-term PD is thought to induce a high-transport state accompanied by peritoneal fibrosis, angiogenesis, and ultimately peritoneal membrane failure. However, histopathological analysis at the endpoint demonstrated that the GEL group showed the mildest increase in peritoneal thickness and collagen deposition (with no statistical difference compared to the NS group). In the GEL-PDF group, the observed trends of a higher BUN D/P ratio and reduced transperitoneal ALB loss align with its preserved peritoneal structure and optimized physiological transport function. This may be linked to its low glucose content, low osmolality, neutral pH, and mild citrate-based buffer system. Further investigation is warranted to determine whether these associations represent a sustained functional benefit.

When analyzing the experiment result after 12 weeks, we were surprised to find that the 4 h effluent volume in the GLU group was exceptionally low. And we further compared the effluent volumes between the short-term experiment and the 12-week time point. Statistical analysis confirmed a catastrophic decline in the UF of the GLU group, dropping from an initial 12.5 ± 3.1 mL to 1.7 ± 1.9 mL (*p* = 0.001, independent samples *t*-test). In contrast, the drainage volume in the GEL group remained largely consistent (*p* = 0.174). This finding further highlights the ability of the GEL-PDF to maintain an effective balance between preserving peritoneal structure and sustaining adequate UF during long-term application. Although GEL-PDF exhibited a lower UF volume than the ICO-PDF during long-term dwell, its combination of moderate yet sustained UF and superior biocompatibility suggests it may be positioned as a potential agent for short- to medium-dwell exchanges. If substantiated, this could address an unmet need for a solution that balances efficacy and tissue compatibility in these frequent exchanges. This proposed role remains speculative and requires further preclinical optimization and clinical validation.

Furthermore, histological examination of renal sections at the end of the 12-week experiment revealed observed variation in renal adenine crystal deposition, with the least accumulation found in the GEL group. This variation is speculatively linked to the composition of the PDF and invites the hypothesis that certain components might modulate crystal deposition or clearance, a possibility that warrants further mechanistic investigation.

In contrast to the conventional lactate buffer (40 mmol/L), we selected citrate at a concentration of 14 mmol/L. This decision was based on two key considerations: its renal-independent metabolic pathway and its potential for enhanced local biocompatibility [15,20,25]. Citrate is primarily metabolized in the liver via the tricarboxylic acid (TCA) cycle to HCO_3_^−^, a process that does not rely on renal function, thereby theoretically avoiding accumulation risks in ESKD patients. The difference in alkali-generating capacity between citrate and lactate was fully considered during formulation design. The theoretical maximum alkali load from 14 mmol/L citrate (where one molecule yields three HCO_3_^−^ molecules) is similar to that from 40 mmol/L lactate. Furthermore, compared to lactate, citrate metabolism involves multiple rate-limiting enzymatic steps, potentially resulting in a slower and more gradual generation of HCO_3_^−^. Moreover, the experimental data showed no significant difference in HCO_3_^−^ levels among the groups at the endpoint (*p* = 0.831), and no risk of metabolic alkalosis was detected. Moreover, the iCa^2+^ concentration in the effluent of the GEL group showed no difference from other groups (*p* = 0.113), and no statistical differences were found in serum total or ionized calcium levels across groups, ruling out concerns of hypocalcemia due to citrate chelation. However, the evidence from this study indicates a favorable safety profile for the low-dose citrate buffer, within the context of a 12-week, once-daily dwell model. Future preclinical studies with longer durations and more frequent exchanges, better simulating clinical dialysis regimens, are warranted to further evaluate its long-term impact.

However, this study has several limitations. First, due to constraints in raw materials, we prepared only one concentration (4%) of the GEL-PDF, preventing the exploration of the relationship between concentration gradients and UF volume. Second, the long-term animal model utilized single daily intraperitoneal injections, which differs from the continuous cyclical nature of clinical PD. Additionally, the daily dosing regimen does not fully replicate the clinical scenario. The relatively small sample size in each group, particularly in the NS group (*n* = 5), may have limited the statistical power to detect more subtle intergroup differences. Third, the adenine-induced CKD model, while well-established, primarily represents a model of progressive tubular injury and fibrosis, and may not fully capture all aspects of the uremic state in human patients on long-term PD. Furthermore, although GEL appeared to preserve serum TP levels, the residual GEL in the effluent significantly interfered with the biuret assay, precluding a direct comparison of TP loss in effluent across groups. Moreover, future studies employing specific assays or tracer techniques are warranted to directly characterize its absorption, distribution, and metabolism, which will further elucidate the mechanistic basis of its sustained intraperitoneal presence and safety profile. Finally, the 12-week study period, while sufficient to demonstrate initial efficacy and safety, is insufficient to evaluate the very long-term biocompatibility and functional integrity of the peritoneum, which evolves over years in human patients.

## 4. Materials and Methods

### 4.1. Preparation of Novel Peritoneal Dialysis Fluids and Basic Information of Control Fluids

The novel GEL-PDF was prepared from 4% succinylated gelatin injection (Gelofusine^®^), supplemented with sodium citrate, calcium chloride, and magnesium chloride (Chinese Patent No. ZL202411472422.9). The detailed formulation is provided in Table 4, and all prepared PDFs underwent autoclaving. Control PDFs included Normal Saline (NS) (Chengdu Qingshan Likang Pharmaceutical, Chengdu, China), 1.5% GLU-PDF (Chengdu Qingshan Likang Pharmaceutical), and ICO-PDF (Baxter Healthcare (Guangzhou, China)).

### 4.2. Short-Term Animal Experiment

**Figure 6:**
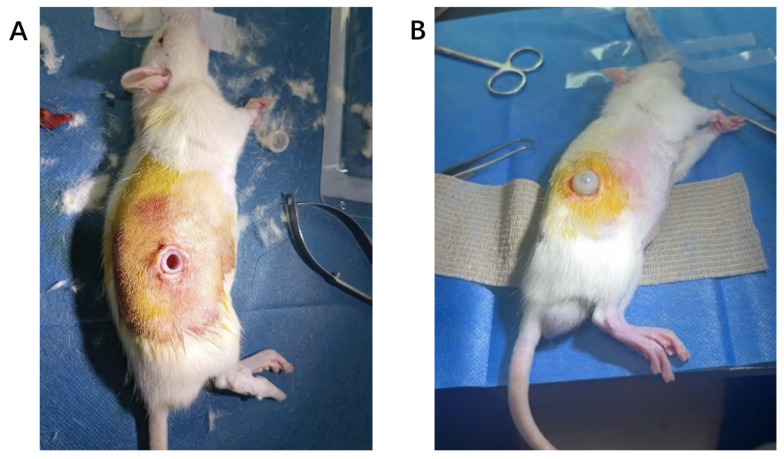
(**A**,**B**) Establishment of peritoneal dialysis model in rats.

A total of 20 male Wistar rats weighing 250–300 g was obtained from Beijing Vital River Laboratory Animal Technology (Beijing, China). A chronic kidney disease (CKD) model was induced in the rats by maintaining them on a 0.5% adenine diet (Anhui Kuibu Shuyu Biotechnology, Hefei, China) for 4 weeks. Under isoflurane anesthesia (Shenzhen Ruiwand Life Science and Technology, Shenzhen, China), a PD catheterization procedure was performed using a custom-made silicone catheter assembly with an external protective sleeve (Figure 6). All animals recovered within 1–2 min post-procedure and were allowed free access to food and tap water. After one-week recovery, PD was initiated. Baseline Scr levels were measured before formal experiment. Under anesthesia, the protective sleeve was aseptically opened, and 30 mL of pre-warmed (37 °C) PDF was injected intraperitoneally. Effluent samples were collected under anesthesia at predetermined dwell time points.

### 4.3. Long-Term Animal Experiment

Thirty-five male Wistar rats weighing 250–300 g were obtained from Beijing Vital River Laboratory Animal Technology. The CKD model was induced using the same protocol as described above. After 4 weeks, serum creatinine (Scr) and other biochemical parameters were measured as the baseline. Five rats were allocated to the NS group, while the remaining thirty rats were evenly distributed among the GLU, ICO, and GEL groups (10 rats per group). The rats then received daily intraperitoneal injections of 30 mL of pre-warmed PDF for 12 weeks, with administration sites alternating between the left and right lower abdomen. Blood samples were collected at 4, 8, and 12 weeks. Terminal sampling was performed under anesthesia: a small abdominal incision was made to collect peritoneal effluent, followed by careful excision of mid-abdominal parietal peritoneum and entire kidneys for subsequent histological examination. Throughout the study, all animals were maintained on a 1:1 mixed diet of 0.5% adenine diet and standard rodent chow. The experimental protocol was approved by the Ethics Committee for Animal Experiments of Chengdu Yibang Medical Technology (Approval No: EBFL-202410005; Approval Date: 5 October 2024). It is important to acknowledge that the once-daily intraperitoneal exposure model used in this study differs from the continuous, cyclic exchanges of standard clinical PD. This simplified regimen was chosen to enable a controlled, preliminary assessment of the novel PDF’s long-term biocompatibility and safety profile. And the relatively small size of the normal saline group (*n* = 5) is a limitation at an earlier stage.

### 4.4. Biochemical Analyses

Scr, blood urea nitrogen (BUN), ionized calcium (iCa^2+^), sodium (Na^+^), chloride (Cl^−^), potassium (K^+^), random blood glucose, and lactate (Lac) were measured using a portable blood gas analyzer (Abbott i-STAT 300, Abbott Rapid, Orlando, FL, USA). Total calcium (Ca^2+^), phosphorus (P), alkaline phosphatase (ALP), aspartate aminotransferase (AST), alanine aminotransferase (ALT), total cholesterol (TC), low-density lipoprotein (LDL), albumin (ALB), total protein (TP), and glycated serum protein (GSP) were analyzed with an automatic biochemistry analyzer (Shenzhen Rayto Life and Technology, Shenzhen, China). For peritoneal effluent samples, Cre, urea nitrogen, iCa, Na, Cl, and K were determined using the portable blood gas analyzer, while the albumin was assessed with the automatic biochemistry analyzer.

### 4.5. Histological Analysis of Peritoneum and Kidney

Peritoneal and renal tissues were processed for histological evaluation. Briefly, samples were fixed in 4% paraformaldehyde, embedded in paraffin, and sectioned at 5 μm thickness. For peritoneal morphology, sections were stained with Hematoxylin and Eosin (H&E) to evaluate general structure and measure peritoneal thickness. The peritoneal thickness was measured at five randomly selected fields per section, and the mean value was calculated to represent the final peritoneal thickness for each rat. Masson’s Trichrome staining was subsequently performed to quantify collagen deposition and calculate the collagen fiber area ratio, assessing the degree of peritoneal fibrosis. Renal sections were stained with H&E to examine adenine crystal deposition, thereby verifying the establishment of the CKD model. All stained sections were examined under a light microscope, and images were analyzed using the ImageJ software (National Institutes of Health, USA, version 1.54p) for quantitative measurements. While quantitative histomorphometry was performed using objective image analysis software, the histological assessor was not formally blinded due to the potential recognizability of treatment-induced morphological changes.

### 4.6. Statistical Analysis

All data are expressed as the mean ± standard deviation (SD). The normality of data distribution was assessed using the Shapiro–Wilk test, and homogeneity of variances was verified using Bartlett’s test. For comparisons among multiple groups meeting both assumptions, one-way analysis of variance (ANOVA) was applied, followed by Tukey’s post hoc test for pairwise comparisons. When the assumptions of normality or equal variance were violated, the non-parametric Kruskal–Wallis (K-W) test was used, followed by Dunn’s test for post hoc analysis. For longitudinal measurements, statistical analyses were performed as cross-sectional comparisons at each individual time point. This approach focuses the analysis on between-group differences at specific stages of the intervention and is supported by the primary objective of comparing long-term outcomes. All statistical analyses were performed using the R software (version 4.2.2). A *p* value of ≤0.05 was considered significant.

## 5. Conclusions

This study developed and evaluated a novel PDF using GEL as osmotic agent and citrate as buffer. Short-term experiment confirmed its effective solute clearance and UF capacity during a 4 h dwell. Long-term investigation further demonstrated that, compared to GLU-PDF, the GEL-PDF exhibited superior biocompatibility, manifested as significantly reduced peritoneal fibrosis and thickening, along with better preservation of serum ALB levels.

Although its long-duration UF efficacy was lower than that of ICO, this characteristic makes it particularly suitable for short-to-medium dwell. Thus, the novel GEL-PDF represents a promising colloidal osmotic agent for daily exchanges, potentially filling the current clinical gap for short-dwell therapy where ICO is not applicable. It offers a new option for preserving peritoneal membrane integrity and optimizing dialysis regimens.

## Figures and Tables

**Table 1:** Physicochemical properties of the peritoneal dialysis fluids used.

	Normal Saline	1.5% Glucose-Based Peritoneal Dialysis Fluid	Icodextrin-Based Peritoneal Dialysis Fluid	Succinylated Gelatin-Based Peritoneal Dialysis Fluid
pH	6.85	5.37	7.05	7.30
Osmolality	263	340	286	317
Osmotic agent	/	1.5% Glucose	7.5% Icodextrin	4% Succinylated Gelatin
Molecular weight (Da)	/	180	13,000–19,000	20,000~37,000
Buffer	/	40 mmol/L Lactate	40 mmol/L Lactate	14 mmol/L Citrate

**Table 2:** Blood biochemical parameters—monitored at the terminal point (sample size: NS:5, GLU:6, ICO:7, GEL:7).

Parameters	NS	GLU	ICO	GEL	*p*
GSP mmol/L	1.10 ± 0.47	1.01 ± 0.33	1.17 ± 0.19	0.97 ± 0.08	0.173
Lactate mmol/L	1.7 ± 0.8	4.0 ± 3.1	2.3 ± 0.8	3.2 ± 1.3	0.150
Random Blood Glucose mmol/L	11.9 ± 4.1	11.6 ± 2.0	12.6 ± 1.9	12.5 ± 1.8	0.866

GSP: glycated serum protein.

**Table 3:** Biochemical parameters in peritoneal effluent after 12 weeks (sample sizes: NS:5, GLU:3 [only three with effluent], ICO:7, GEL:7).

Parameters	NS	GLU	ICO	GEL	*p*	Notes
Effluent Volume mL	10.0 ± 4.6	2.3 ± 1.2	48.0 ± 4.9	28.6 ± 8.1	<0.001	GLU—GEL *p* = 0.017 GLU—ICO *p* < 0.001 NS—ICO *p* = 0.007
iCa^2+^ mmol/L	1.25 ± 0.05	1.31 ± 0.02	1.33 ± 0.05	1.28 ± 0.07	0.113	
Na^+^ mmol/L	143 ± 3	143 ± 2	142 ± 5	144 ± 2	0.340	
Cl^−^ mmol/L	105 ± 1	102 ± 2	103 ± 7	102 ± 1	0.077	
K^+^ mmol/L	4.9 ± 0.5	4.5 ± 0.3	4.3 ± 0.4	4.4 ± 0.3	0.077	NS—ICO *p* = 0.030
Albumin g/L	6.2 ± 3.0	6.0 ± 1.8	5.2 ± 1.5	4.5 ± 2.4	0.507	
Creatinine D/P	0.99 ± 0.07	0.98 ± 0.05	0.97 ± 0.16	0.94 ± 0.09	0.847	
Urea Nitrogen D/P	0.77 ± 0.04	0.92 ± 0.14	0.94 ± 0.13	0.99 ± 0.18	0.081	NS—GEL *p* = 0.062

**Table 4:** Composition of succinylated gelatin peritoneal fluid (per liter).

Component	Amount
Succinylated gelatin (g/L)	35–40
Sodium citrate (mmol/L)	0.5–0.6
Magnesium citrate (mmol/L)	0.2–0.4
Citrate (mmol/L)	11–13
Sodium (mmol/L)	130–135
Calcium (mmol/L)	0.25–0.5
Magnesium (mmol/L)	0–0.1
Chloride (mmol/L)	108–112
Glucose (mmol/L)	4–5.5

## Data Availability

The original contributions presented in this study are included in the article/Supplementary Materials. Further inquiries can be directed to the corresponding author.

## Supplementary Materials

The following supporting information can be downloaded at https://www.mdpi.com/article/10.3390/ph19020222/s1; Table S1: Blood Biochemical Parameters—Monitored Every 4 Weeks; Table S2: Total Protein levels in GEL-PDF effluent (*n* = 5 for each time points); Figure S1: Total Protein levels in GEL-PDF effluent (*n* = 5 for each time points).

## Abbreviations

ALB	Albumin
ALP	Alkaline Phosphatase
ALT	Alanine Aminotransferase
AST	Aspartate Aminotransferase
BUN	Blood Urea Nitrogen
CKD	Chronic Kidney Disease
Cl^−^	Chloride ion
Cre	Creatinine
D/P	Dialysate-to-Plasma ratio
ESKD	End-Stage Kidney Disease
GEL	Gelatin-based PDF/Gelofusine
GFR	Glomerular Filtration Rate
GLP	Glycated Plasma Protein
GLU	Glucose-based PDF
iCa^2+^	Ionized Calcium
ICO	Icodextrin-based PDF
K^+^	Potassium ion
Lac	Lactate
LDL	Low-Density Lipoprotein
Na^+^	Sodium ion
NS	Normal Saline
PD	Peritoneal Dialysis
PDF	Peritoneal Dialysis Fluid
RRT	Renal Replacement Therapy
Scr	Serum Creatinine
TC	Total Cholesterol
TP	Total Protein
UF	Ultrafiltration
