# Supplementary material for: A Novel Peritoneal Dialysis Fluid Based on Succinylated Gelatin and Citrate: A Preliminary Investigation of Efficacy, Safety, and Biocompatibility
Source: Pharmaceuticals (Basel). 2026 Jan 27;19(2):222. doi: 10.3390/ph19020222 (PMC12942898; doi:10.3390/ph19020222)
Supplement: Supplementary file 1 [file pharmaceuticals-19-00222-s001.zip › pharmaceuticals-4095081-supplementary.pdf]

Table S1. Blood Biochemical Parameters—Monitored Every 4 Weeks

Parameters		NS<br>Baseline: n=5<br>4 weeks: n=5<br>8 weeks: n=5<br>12 weeks: n=5	GLU<br>Baseline: n=10<br>4 weeks: n=7<br>8 weeks: n=7<br>12 weeks: n=6	ICO<br>Baseline: n=10<br>4 weeks: n=10<br>8 weeks: n=10<br>12 weeks: n=7	GEL<br>Baseline: n=10<br>4 weeks: n=9<br>8 weeks: n=8<br>12 weeks: n=7	P	Notes
Scr mg/dL	Baseline	49.6±8.3	50.1±9.2	50.8±6.3	50.4±12.2	0.996	
	4 weeks	54.2±9.5	54.9±9.1	46.5±10.4	39.0±4.8	0.010	Glu – Gel P=0.004<br>Gel – NS P=0.023
	8 weeks	60.0±4.9	50.7±8.3	45.5±10.9	42.0±10.8	0.029	Gel – NS P=0.017<br>ICO – NS P=0.044
	12 weeks	57.4±4.2	50.2±8.1	51.3±9.5	47.4±4.1	0.126	
BUN mg/dL	Baseline	32.8±5.5	30.8±6.4	29.4±3.3	29.6±8.8	0.769	
	4 weeks	31±5.4	24.9±4.3	25.7±4.4	17.1±3.1	< 0.001	NS – Gel P < 0.001<br>Glu – Gel P=0.021<br>ICO – Gel P=0.006
	8 weeks	26.0±4.9	24.7±6.0	23.7±4.1	18.5±2.5	0.017	ICO – Gel P=0.078<br>NS – Gel P=0.026<br>Gel – Glu P=0.062
	12 weeks	26.2±7.3	16.3±3.6	19.1±7.2	21.6±2.8	0.045	Glu – NS P=0.024
ALB g/L	Baseline	35.1±2.6	34.4±2.9	35.1±3.1	35.4±3.1	0.888	
	4 weeks	35.9±0.8	35.2±2.3	37.2±2.4	35.2±2.1	0.159	
	8 weeks	34.9±1.2	35.9±2.0	35.8±1.5	34.4±2.5	0.410	
	12 weeks	28.8±1.6	28.8±1.1	33.5±2.3	34.1±2.7	< 0.001	NS – ICO P=0.011<br>NS – GEL P=0.002<br>Glu – ICO P=0.007

							Glu – GEL P=0.001
Total Protein g/L	Baseline	54.2±4.1	54.8±2.9	54.6±3.1	53.3±2.4	0.662	
	4 weeks	56.6±2.8	60.5±4.0	62.1±3.0	59.7±2.2	0.024	NS – ICO P=0.014
	8 weeks	53.6±2.2	60.6±2.0	58.2±1.3	57.9±2.0	< 0.001	NS – ICO P < 0.001<br>NS – GEL P=0.002<br>Glu – NS P < 0.001
	12 weeks	43.3±3.2	46.8±2.3	52.0±4.9	50.6±2.4	0.001	NS – ICO P=0.001<br>NS – GEL P=0.008<br>Glu – ICO P=0.056
ALP U/L	Baseline	192±36.8	189±29.9	198±21.9	189±18.0	0.835	
	4 weeks	261±43.2	280±29.6	222±49.0	174±41.1	< 0.001	NS – GEL P < 0.001<br>Glu – GEL P=0.004
	8 weeks	164±25.8	151±12.1	129±15.3	124±14.9	< 0.001	NS – ICO P=0.005<br>NS – GEL P=0.002<br>Glu – GEL P=0.030
	12 weeks	135±10.9	128±22.3	120±25.9	103±19.7	0.076	NS – GEL P=0.078
AST U/L	Baseline	82.5±26.9	83.1±11.5	79.2±5.9	85.0±7.0	0.248	
	4 weeks	87.0±13.9	108.4±20.8	119.1±15.9	116.0±13.0	0.008	NS – ICO P=0.028<br>NS – GEL P=0.025
	8 weeks	77.0±13.0	103.3±23.6	106.0±20.8	100.8±9.3	0.051	NS – ICO P=0.055<br>NS – GEL P=0.076<br>NS – Glu P=0.041
	12 weeks	87.0±20.6	81.4±7.1	85.1±22.8	89.7±7.0	0.475	
ALT U/L	Baseline	26.4±8.99	30.0±7.19	28.0±6.06	27.7±6.2	0.77	
	4 weeks	43.6±7.47	47.1±11.5	44.5±8.13	37.8±5.34	0.153	
	8 weeks	43.5±5.91	41.8±7.95	47.8±5.34	38.2±9.76	0.076	ICO – GEL P=0.052

	12 weeks	38.8±4.3	31.2±4.9	36.3±12.1	32.8±5.6	0.368	
LDL mmol/L	Baseline	0.4±0.1	0.3±0.1	0.3±0.1	0.3±0.1	0.652	
	4 weeks	0.3±0.0	0.3±0.1	0.4±0.1	0.3±0.1	0.429	
	8 weeks	0.3±0.1	0.3±0.1	0.4±0.1	0.4±0.1	0.095	
	12 weeks	0.3±0.1	0.3±0.1	0.3±0.1	0.4±0.2	0.442	
TC mmol/L	Baseline	3.8±1.0	3.3±0.7	3.5±0.4	3.6±0.3	0.483	
	4 weeks	3.6±0.5	2.3±0.1	2.5±0.4	2.0±0.3	< 0.001	ICO – GEL P=0.049<br>NS – GEL P < 0.001<br>NS – Glu P < 0.001<br>NS – ICO P < 0.001
	8 weeks	3.1±0.6	2.1±0.3	2.6±0.5	2.2±0.1	0.004	NS – GEL P < 0.001<br>NS – Glu P < 0.001
	12 weeks	2.5±0.5	2.0±0.2	2.6±0.9	1.8±0.4	0.007	ICO – GEL P=0.035<br>NS – GEL P=0.018
iCa^{2+} mmol/L	Baseline	1.18±0.08	1.26±0.07	1.23±0.09	1.21±0.06	0.202	
	4 weeks	1.34±0.02	1.36±0.04	1.37±0.05	1.35±0.05	0.750	
	8 weeks	1.32±0.04	1.31±0.11	1.34±0.03	1.35±0.05	0.432	
	12 weeks	1.28±0.07	1.26±0.07	1.22±0.06	1.17±0.17	0.184	
Ca^{2+} mmol/L	Baseline	3.2±0.1	3.0±0.2	3.0±0.2	3.0±0.2	0.138	
	4 weeks	3.0±0.2	3.2±0.1	3.1±0.2	3.0±0.2	0.074	
	8 weeks	2.9±0.1	2.8±0.1	2.9±0.1	2.9±0.2	0.209	
	12 weeks	2.9±0.2	3.0±0.3	3.0±0.1	2.9±0.2	0.566	
Na^{+} mmol/L	Baseline	140±4	141±4	140±2	139±2	0.413	
	4 weeks	140±1	142±2	141±2	140±2	0.056	Glu – GEL P=0.040
	8 weeks	141±2	143±2	140±1	142±2	0.050	Glu – ICO P=0.040

	12 weeks	140±3	142±1	142±6	142±2	0.707	
Cl^- mmol/L	Baseline	103±3	100±2	99±2	101±2	0.052	NS – ICO P=0.032
	4 weeks	105±3	105±2	102±2	103±2	0.007	NS – ICO P=0.015<br>Glu – ICO P=0.031
	8 weeks	104±2	106±1	106±1	103±2	0.008	Glu – GEL P=0.047<br>ICO – GEL P=0.007
	12 weeks	105±2	103±1	103±4	103±3	0.527	
K^+ mmol/L	Baseline	4.2±0.4	4.0±0.4	4.1±0.3	4.6±0.2	< 0.001	Glu – GEL P < 0.001<br>ICO – GEL P=0.010<br>NS – GEL P=0.098
	4 weeks	4.1±0.4	4.1±0.4	4.2±0.3	4.3±0.2	0.386	
	8 weeks	4.3±0.3	3.8±0.2	4.2±0.4	4.2±0.2	0.090	
	12 weeks	4.1±0.2	3.8±0.2	3.9±0.3	3.8±0.3	0.181	
P mmol/L	Baseline	2.89±0.72	2.89±0.53	2.88±0.22	2.90±0.20	0.999	
	4 weeks	2.61±0.51	2.76±0.49	2.61±0.28	2.62±0.36	0.859	
	8 weeks	2.32±0.18	2.13±0.12	2.12±0.23	2.21±0.34	0.468	
	12 weeks	2.78±0.55	2.24±0.13	2.14±0.14	2.23±0.52	0.050	NS – GEL P=0.047<br>NS – ICO P=0.034

Scr: serum creatinine; BUN: blood urea nitrogen; ALB: albumin; ALP: alkaline phosphatase; AST: aspartate aminotransferase; ALT: alanine aminotransferase; TC: total cholesterol; LDL: low-density lipoprotein.

Table S2. Total Protein levels in GEL-PDF effluent (n=5 for each time points)

Time	Total protein (g/L)
t=0h	36.0
t=15min	33.6±0.98
t=30min	32.4±1.25
t=60min	30.5±1.69
t=120min	29.1±2.46
t=240min	27.4±1.88
t=480min	23.5±1.54

Figure S1. Total Protein levels in GEL-PDF effluent (n=5 for each time points)

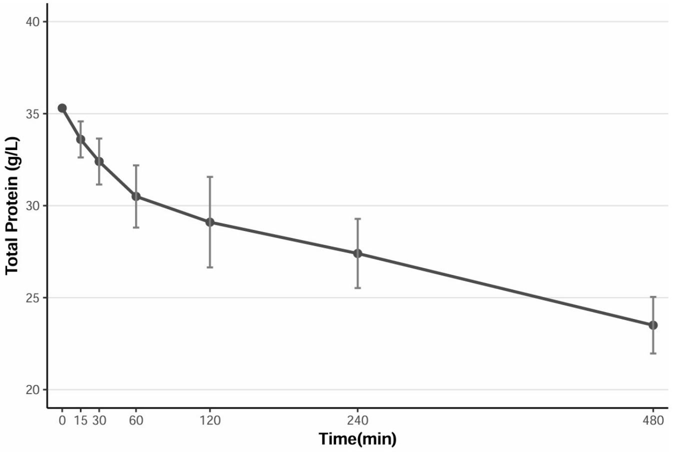

Given that GEL is a hydrolyzed collagen derivative whose backbone consists of peptide bonds, the biuret method—which detects peptide bonds generically—can provide an indirect measure of GEL-related polypeptides in the effluent. While the biuret method cannot detect the smallest terminal degradation products of GEL, such as dipeptides, it can detect polypeptide fragments containing at least two peptide bonds. Therefore, the "total protein" level in the peritoneal effluent can be roughly considered indicative of the GEL content.
